# One-Pot Encapsulation of a Robust α-L-Rhamnosidase Mutant in Cu-MOF: A Recyclable Designed Biocatalyst for High-Yield Conversion of Icariin to Icariside I

**DOI:** 10.3390/molecules31203615

**Published:** 2026-10-10

**Authors:** Haoyu Jia, Tong Yan, Luran Wang, Lingling Song

**Affiliations:** School of Pharmacy, Henan University of Chinese Medicine, Zhengzhou 450046, China; 15137518359@163.com (T.Y.); 16639293199@163.com (L.W.); 18838221762@163.com (L.S.)

**Keywords:** icariside I, α-L-Rhamnosidase mutant, enzyme immobilization, Cu-based MOF, enzyme recyclability

## Abstract

Icariside I is a pharmacologically important prenylated flavonoid glycoside, yet its efficient biocatalytic production from icariin is hindered by the limited stability and recyclability of the enzymes involved. In this study, an α-L-rhamnosidase mutant R783A (Rha R783A) was heterologously expressed, purified, and immobilized within a Cu-based metal–organic framework (MOF) via a one-pot co-precipitation strategy. The resulting Rha R783A@MOF biocomposite exhibited enhanced stability compared to the free enzyme, retaining >90% residual activity after incubation at 60 °C for 2 h and 86.4% after 30 days of storage. It also demonstrated broad pH adaptability. In the biotransformation of icariin, Rha R783A@MOF achieved a 99.8% conversion rate and a 97.8% yield of icariside I. Furthermore, the biocomposite maintained an 88.9% yield after eight consecutive reuse cycles under high-temperature conditions (60 °C), demonstrating excellent operational stability. This work presents a robust and sustainable strategy for icariside I production, highlighting the potential of MOF microparticles as an effective platform for the biocatalytic synthesis of high-value flavonoid compounds.

## 1. Introduction

α-L-Rhamnosidase (EC 3.2.1.40) is a glycoside hydrolase that specifically catalyzes the cleavage of terminal α-L-rhamnose residues from a wide range of natural products, including flavonoids, terpenoids, and steroids [1,2]. This unique substrate specificity has positioned the enzyme as a pivotal biocatalyst in the food processing, pharmaceutical, and biotransformation industries [3,4,5]. One of the most promising applications of α-L-rhamnosidase is the enzymatic conversion of icariin, a major bioactive flavonoid from *Herba epimedii*, into icariside I, a deglycosylated derivative with significantly enhanced pharmacological properties, including improved bioavailability, antioxidant, and anti-inflammatory activities [6,7,8,9]. Although icariin is abundant than icariside I in traditional Chinese medicinal herbs, its poor oral bioavailability limits therapeutic efficacy; enzymatic removal of the terminal rhamnose moiety yields icariside I, which exhibits superior solubility and cellular uptake. Recent studies have demonstrated that α-L-rhamnosidase from *Dictyoglomus thermophilum* (DthRha) can achieve over 92% conversion of icariin to icariside I under optimized conditions, highlighting the great potential of this enzymatic approach [5].

However, the industrial application of free α-L-rhamnosidase is severely constrained by several intrinsic limitations. The enzyme typically exhibits poor operational stability, susceptibility to denaturation under harsh reaction conditions, difficulty in recovery and reuse, and challenges in product separation. These drawbacks not only increase the cost of biocatalytic processes but also hinder the scalable production of high-value flavonoid glycosides. To overcome these bottlenecks, enzyme immobilization has emerged as a cornerstone strategy that anchors enzymes onto solid supports, thereby improving stability, enabling facile recovery and repeated use, and simplifying downstream processing [10,11,12]. The physicochemical properties of the support, including specific surface area, porosity, surface functionality, and biocompatibility, directly determine the catalytic efficiency and operational performance of the resulting immobilized biocatalyst.

Among the diverse support materials, metal–organic frameworks (MOFs) have attracted significant attention as promising immobilization matrices due to their exceptionally high surface areas, tunable pore structures, and versatile chemical functionalities [13,14]. Recent studies have demonstrated the significant potential of MOF-based biocomposites in flavonoid and glycoside biotransformation. For instance, Ogunbadejo et al. immobilized cyclodextrin glycosyltransferase on hierarchical Cu-based MOF, achieving 87% activity retention after ten cycles for cyclodextrin production [15]. Similarly, β-glucosidase immobilized on Cu-based MOF via one-pot co-precipitation achieved 99.8% conversion of icariin to baohuoside I and retained 70.5% activity after eight cycles [16]. In addition, Li et al. immobilized glucosyltransferases on mesoporous Zn-Ni MOFs through metal chelation, enabling one-step enzyme purification and efficient catalytic conversion of ginsenoside Rh2, with 76% residual activity after seven cycles and 80.22% activity after 45 days of storage [17]. Therefore, MOF-based enzyme immobilization represents a versatile and promising strategy for enhancing the stability, reusability, and overall catalytic performance of biocatalysts in the production of high-value bioactive natural products.

Despite these advances, the development of an efficient, recyclable biocatalytic system for the conversion of icariin to icariside I remains a significant challenge. Although the free α-L-rhamnosidase from *Dictyoglomus thermophilum* exhibited excellent organic solvent tolerance and catalytic efficiency, the rational design and systematic evaluation of an immobilized enzyme system for this biotransformation have yet to be explored. To address this challenge, a recyclable Rha R783A@MOF biocomposite by one-pot encapsulation of the α-L-rhamnosidase mutant R783A (Rha R783A) within a copper-based MOF was developed. The immobilization conditions were systematically optimized, and the resulting biocomposite was comprehensively characterized to elucidate its structural and catalytic properties. Furthermore, its catalytic performance, operational stability, and reusability were evaluated for the conversion of icariin to icariside I. This work provides an efficient and sustainable immobilized biocatalytic platform for the industrial production of the high-value bioactive compound icariside I.

## 2. Results and Discussion

### 2.1. Expression and Purification of Recombinant α-L-Rhamnosidase R783A

The wild-type α-L-rhamnosidase gene from *Dictyoglomus thermophilum* (GenBank: WP_012548615) was cloned and recombinantly expressed in the pET-28a(+) vector. Due to the limited activity and stability of the wild-type enzyme, site-directed mutagenesis was performed in our previous work [18], among which the Rha R783A variant exhibited a 3.56-fold increase in enzymatic activity relative to the wild-type enzyme (WT), along with remarkable thermal stability, retaining over 92% of its initial activity after 2 h incubation at 60–90 °C. To improve the reusability of the enzyme, the α-L-rhamnosidase R783A variant was therefore selected for immobilization in this work.

To obtain sufficient purified α-L-rhamnosidase R783A for subsequent immobilization, the recombinant enzyme was heterologously expressed in *Escherichia coli* BL21(DE3) using the pET-28a expression plasmid. The recombinant plasmid carrying the α-L-rhamnosidase R783A gene was successfully introduced into the host strain, and protein expression was induced by IPTG. The expressed protein was subsequently purified by Ni-NTA affinity chromatography through the N-terminal His-tag fusion. The His-tag was retained for purification and had no significant influence on the catalytic performance of the enzyme [5]; since the enzyme was expressed in *E. coli*, it is not glycosylated. SDS-PAGE analysis revealed a distinct band corresponding to the expected molecular mass of approximately 102 kDa (Figure 1), confirming the successful expression of the recombinant enzyme. The successful expression and purification of α-L-rhamnosidase R783A provided a reliable source of enzyme for subsequent immobilization studies.

### 2.2. Preparation of Rha R783A@MOF and Optimization of Immobilization Parameters

The Rha R783A@MOF biocomposite was successfully fabricated via one-pot co-precipitation encapsulation strategy (Scheme 1), in which the enzyme was entrapped within the Cu-based MOF matrix during framework crystallization. To maximize the immobilization efficiency and catalytic performance of the resulting biocatalyst, three key parameters, including enzyme concentration, PVP concentration, and immobilization time, were systematically optimized by monitoring enzyme loading and relative activity. As shown in Figure 2, the enzyme loading increased progressively with increasing Rha R783A concentration from 0.1 to 1.0 mg/mL, reaching a relatively high value of 123.3 mg/g at 1.0 mg/mL, beyond which no significant increase was observed. Notably, the relative activity of Rha R783A@MOF also reached its optimum at an enzyme concentration of 1.0 mg/mL, indicating that this concentration provided sufficient enzyme loading. PVP modifies the enzyme surface to strengthen its interactions with metal ions and organic linkers, thereby improving the encapsulation efficiency [19]. Meanwhile, the pyrrolidone groups of PVP weakly coordinate with metal cations to enrich them on the enzyme surface, promoting MOF nucleation and crystal growth around the enzyme [20]. When the PVP concentration was varied from 0.1 to 2.0 mg/mL, the enzyme loading increased steadily up to 148.9 mg/g, with further increases resulting in a gradual decline in both loading and relative activity. Therefore, 0.5 mg/mL was identified as the optimal PVP concentration. Variations in PVP chain length may affect enzyme loading capacity and the accessibility of active sites, which could warrant further optimization. The immobilization time was optimized by balancing enzyme loading and activity retention. Relative activity increased with time up to 8 h, beyond which it decreased, despite enzyme loading reaching its maximum (159.6 mg/g) at 10 h. The decline in activity after 8 h may result from extended exposure to the synthesis environment, which could constrain the conformational dynamics required for catalysis. Consequently, 8 h was chosen as the optimal immobilization time to ensure both satisfactory loading and maximal catalytic activity.

### 2.3. Characterization of MOF and Rha R783A@MOF

The morphologies of the pristine MOF and Rha R783A@MOF biocomposite were characterized by SEM. As shown in Figure 3a, the pristine MOF exhibited flower-like particles with diameters of approximately 4 μm. After Rha R783A encapsulation, the overall particle size remained nearly unchanged (Figure 3b). However, the Rha R783A@MOF biocomposite displayed more pronounced flower-like structures and a slightly higher degree of aggregation than the pristine MOF [21]. This is likely because the exposed amino or carboxyl groups on the enzyme surface enrich metal ions and accelerate the formation of prenuclear clusters around the enzyme, thereby promoting MOF nucleation and encapsulation. The distribution of Rha R783A within the composite was further investigated by SEM-EDS elemental mapping. As shown in Figure 3c, no obvious Rha R783A aggregates were observed on the external surface of the biocomposite, while the elemental maps showed a homogeneous distribution of Cu, C, N, O and S throughout the composite. These observations suggested that Rha R783A was uniformly dispersed within the composite, without obvious phase separation or surface aggregation. In particular, the uniform distribution of S, which was present in the enzyme but absent from the MOF and PABA ligand, supported the encapsulation of Rha R783A within the MOF pores rather than simple adsorption onto the external surface.

XRD patterns were employed to evaluate the structural integrity of MOF before and after Rha R783A immobilization (Figure 4a). The characteristic diffraction peaks of MOF remained unchanged after the introduction of Rha R783A, indicating that the immobilization process did not disrupt the original coordination framework. The absence of additional diffraction peaks further suggested that no new crystalline phases were generated during modification. Nevertheless, a slight reduction in diffraction peak intensity was observed for Rha R783A@MOF, this decrease in peak intensity may be attributed to the interaction between Rha R783A molecules and the MOF during the encapsulation process [22].

FTIR analysis was performed to investigate the chemical characteristics of Rha R783A, MOF, and Rha R783A@MOF biocomposite (Figure 4b). The FTIR spectrum of Rha R783A exhibited characteristic absorption bands at approximately 1660 cm^−1^ and 1575 cm^−1^, which were assigned to the C=O stretching vibration of the amide I band and the N-H bending/C-N stretching vibrations of the amide II band, respectively. The amide I band of Rha R783A@MOF appeared at a similar wavenumber to that of free Rha R783A, suggesting no major structural disruption [23]. In addition, the band at around 1425 cm^−1^ was attributed to N-H bending and C-N stretching vibrations, further confirming the characteristic functional groups of Rha R783A [24]. The FTIR spectrum of pristine MOF displayed typical absorption bands associated with its framework structure. The peak at approximately 472 cm^−1^ was assigned to Cu-related vibrations, while the bands located at 1608 and 1390 cm^−1^ were attributed to the stretching vibrations of carboxyl groups and aromatic ring-related vibrations, respectively [25]. These characteristic peaks confirmed the successful formation of the MOF. After incorporation of Rha R783A, the FTIR spectrum of Rha R783A@MOF retained the characteristic peaks of pristine MOF, indicating that the crystal framework of MOF remained intact during the encapsulation process. Meanwhile, the appearance of the amide I band at approximately 1660 cm^−1^ in Rha R783A@MOF confirmed the successful introduction of Rha R783A into the MOF matrix. Since the amide I band is closely associated with the secondary structure of proteins, deconvolution analysis of the amide I region was performed. The contents of α-helix, β-sheet, β-turn, and random coil in Rha R783A@MOF changed by less than 3 percentage points compared with free R783A (Table S1), with a slight decrease in α-helix and a slight increase in β-sheet, indicating that the secondary structure of Rha R783A was largely preserved during immobilization. Compared with the individual components, the characteristic peaks of Rha R783A@MOF exhibited a slight reduction in intensity, which was likely attributed to the shielding effect caused by the encapsulation of Rha R783A within the MOF [26]. These results demonstrated the successful preparation of the Rha R783A@MOF biocomposite while maintaining the structural integrity of MOF.

TGA was performed to verify the successful immobilization of Rha R783A into MOF (Figure 4c). Free Rha R783A displayed two major weight-loss stages: a minor loss below 150 °C due to water evaporation, and a significant loss between 250 and 350 °C corresponding to protein decomposition, with a total weight loss of about 60%. In contrast, pristine MOF exhibited negligible weight loss (~5%) below 150 °C. The Rha R783A@MOF composite showed a weight-loss profile similar to that of MOF at low temperatures, but with an additional decomposition step between 250 and 350 °C, confirming the presence of immobilized Rha R783A.

To further verify that Rha R783A was encapsulated within the MOF crystalline shell rather than merely adsorbed onto its external surface, an equal amount of pure MOF and Rha R783A protein was physically mixed to obtain the Rha R783A&MOF control sample (Figure S1a). The Bradford assay revealed that only 6.6% of R783A was adsorbed onto the outer surface of MOF, whereas the encapsulation efficiency of Rha R783A@MOF reached 84.9%, indicating that Rha R783A was predominantly incorporated into the MOF. Furthermore, after three consecutive washing cycles with SDS solution, only 2.3% of Rha R783A remained in the Rha R783A&MOF sample, while the enzyme loading of Rha R783A@MOF showed negligible variation compared with the unwashed sample (Figure S1b). These characterization results further confirm that Rha R783A was encapsulated within the MOF crystalline shell, rather than merely adsorbed onto its surface.

### 2.4. Catalytic Performance of Immobilized Rha R783A@MOF

#### 2.4.1. Optimization of Catalytic Conditions and Stability Assessment

Reaction pH and temperature are critical factors that significantly influence enzymatic activity. To maximize the catalytic performance of Rha R783A@MOF, the effects of reaction pH and temperature on its catalytic activity were systematically evaluated. As shown in Figure 5a, both free Rha R783A and Rha R783A@MOF exhibited similar pH-dependent activity profiles. Their catalytic activities gradually increased with increasing pH, reached a maximum at pH 8.0, and subsequently declined under more alkaline conditions. Compared with the free enzyme, Rha R783A@MOF retained slightly higher relative activity at pH 6 and 7, reaching 88.4% and 97.4%, respectively, versus 79.8% and 92.7% for the free enzyme, indicating that the one-pot co-precipitation process did not compromise the catalytic properties of Rha R783A. This slight improvement may be partially attributed to the MOF matrix acting as a protective shell. The effect of temperature on the catalytic activity of free Rha R783A and Rha R783A@MOF was shown in Figure 5b. Free Rha R783A exhibited its maximum activity at 70 °C, whereas the optimum temperature of Rha R783A@MOF shifted to 80 °C. Notably, the immobilized enzyme retained more than 90.3% of its maximum activity over a broad temperature range of 60–90 °C, demonstrating markedly enhanced thermal tolerance. In contrast, the activity of free Rha R783A decreased sharply when the temperature exceeded 70 °C, suggesting pronounced thermal inactivation at elevated temperatures. The Rha R783A@MOF biocomposite exhibited improved relative activity compared with the free enzyme under the tested conditions. This improvement may be associated with the presence of the MOF, although the underlying mechanism requires further investigation [27,28].

pH and temperature stability are crucial parameters determining the practical applicability of enzymes in biocatalytic processes. As illustrated in Figure 5c, the pH stability of free Rha R783A and Rha R783A@MOF was evaluated by measuring their residual activities after incubation in buffers with different pH values for 2 h. Both free Rha R783A and Rha R783A@MOF suffered from substantial activity loss under acidic conditions (pH 3.0–5.0), retaining less than 50% of their initial activities, while only minor differences were observed between the two samples. However, when the pH exceeded 5.0, Rha R783A@MOF gradually exhibited higher residual activity than free Rha R783A over the pH range of 5.0–9.0, indicating that immobilization within the MOF effectively enhanced the resistance of Rha R783A against pH-induced deactivation.

Thermal stability is another essential factor for the industrial application of enzymes, as elevated operating temperatures can suppress microbial contamination and improve the solubility and mass transfer of poorly water-soluble substrates [29]. Considering that Rha R783A originates from a thermophilic microorganism, both free Rha R783A and Rha R783A@MOF exhibited excellent intrinsic thermal stability. As shown in Figure 5d, after incubation at 60 °C for 2 h, both free Rha R783A and Rha R783A@MOF retained more than 90% of their initial activity, demonstrating remarkable resistance to thermal inactivation. The immobilized enzyme showed slightly higher residual activity than the free enzyme, retaining 95.2% versus 90.1% for the free enzyme.

The storage stability of free Rha R783A and Rha R783A@MOF was evaluated by monitoring their residual activities during storage at 4 °C (Figure S2). After 20 days of storage, both free Rha R783A and Rha R783A@MOF retained more than 93.2% of their initial activities, indicating excellent storage stability. After prolonged storage for 30 days, Rha R783A@MOF maintained 86.4% of its initial activity, whereas free R783A retained 82.0%. The slightly higher residual activity of Rha R783A@MOF after extended storage was likely attributed to the protective effect of the MOF matrix [30,31]. Similar observations were reported by Wang et al., who encapsulated β-glucosidase in a Cu-MOF through a co-precipitation approach. The immobilized β-glucosidase retained nearly 90% of its initial activity after 30 days of storage [32].

#### 2.4.2. Tolerance to Metal Ions and Organic Solvents

The resistance of free Rha R783A and Rha R783A@MOF to different chemical additives was investigated (Figure 6a). The presence of most tested metal ions caused less pronounced inhibition toward the immobilized biocatalyst than toward the free enzyme. In particular, Rha R783A@MOF exhibited a certain degree of activation in the presence of Fe^2+^ and Fe^3+^. A similar protective effect was observed in the presence of Cu^2+^ and Zn^2+^, both of which are recognized as potent inhibitors of α-L-rhamnosidase [33,34,35]. This enhanced resistance was likely attributable to the porous architecture and abundant adsorption sites of the MOF matrix. Although 1 mM Cu^2+^ inhibited the free enzyme by 23%, the Rha R783A@MOF biocomposite prepared in 50 mM copper acetate retained 91.3% of its activity relative to free Rha R783A (Figure S3). This slight inhibition was attributed to the rapid consumption of Cu^2+^ through coordination with organic linkers, thereby minimizing its adverse impact on the enzyme. Moreover, the inhibition of mutant R783A by 1 mM of Cu^2+^ is partially reversible (Table S2). In contrast, the addition of other tested metal ions resulted in only slight variations in catalytic performance. Furthermore, the activities of both free Rha R783A and Rha R783A@MOF remained largely unchanged in the presence of DTT and EDTA, suggesting that Rha R783A does not require disulfide bond stabilization or metal cofactors for catalytic function under the experimental conditions.

The influence of different organic solvents on the catalytic behavior of free Rha R783A and Rha R783A@MOF was examined (Figure 6b). The enzyme activity remained largely unaffected in the presence of methanol and ethanol, while ACN caused a moderate decrease in catalytic performance. Interestingly, both free and immobilized enzymes exhibited enhanced activity in the presence of DMSO, reaching 139.7% and 144.9% of their initial activities, respectively. This phenomenon agrees with our previous observation that wild-type α-L-rhamnosidase possesses favorable tolerance toward organic solvents [5]. The improved solvent resistance of Rha R783A@MOF in DMSO may facilitate enzymatic conversion of hydrophobic flavonoid substrates by providing a more compatible catalytic microenvironment.

#### 2.4.3. Kinetic Parameter Analysis

The apparent kinetic parameters of free Rha R783A and Rha R783A@MOF were determined according to the Michaelis–Menten model. As shown in Table 1 and Figure S4, compared with free Rha R783A, Rha R783A@MOF exhibited slightly higher catalytic performance. Compared with free Rha R783A, the *V*_max_ of Rha R783A@MOF increased from 2.71 mM s^−1^ to 2.82 mM s^−1^, this difference may be related to the MOF microenvironment. The *K*_m_ value slightly increased from 0.41 mM to 0.48 mM after encapsulation, suggesting a marginal decrease in apparent substrate affinity. This increase may be attributed to restricted substrate diffusion within the MOF channels and partial shielding of enzyme active sites caused by spatial confinement, which together result in increased mass transfer resistance during the catalytic process [36,37]. Nevertheless, the *k*_cat_ value increased from 17.31 s^−1^ for free Rha R783A to 23.05 s^−1^ for Rha R783A@MOF, corresponding to an approximately 33.2% improvement in catalytic turnover. As a result, the catalytic efficiency (*k*_cat_/*K*_m_) increased from 42.22 s^−1^ mM^−1^ to 48.02 s^−1^ mM^−1^, representing an approximately 11.8% enhancement. These results demonstrated that encapsulation within the MOF structure effectively maintained and slightly enhanced the catalytic activity of Rha R783A, which may be related to the MOF microenvironment. Compared with free R783A, Rha R783A@MOF showed slightly higher catalytic turnover despite substrate accessibility was slightly affected.

### 2.5. Application and Reusability of Rha R783A@MOF in Biocatalytic Transformation

To assess the practical applicability of Rha R783A@MOF in biocatalytic transformation, the conversion of icariin into icariside I was investigated. As monitored by HPLC analysis, both catalytic systems efficiently promoted the selective hydrolysis of the rhamnose moiety at the C-3 position of icariin, leading to the formation of icariside I as the predominant product (Figure S5). The time-dependent conversion profiles were presented in Figure 7a. At the initial stage of the reaction, free R783A displayed a slightly faster conversion rate than Rha R783A@MOF within the first hour, which could be attributed to the unrestricted contact between the free enzyme and substrate molecules [30]. However, with prolonged incubation, the catalytic activity of free Rha R783A gradually decreased, whereas Rha R783A@MOF exhibited a more sustained catalytic performance. After 5 h of reaction, Rha R783A@MOF achieved 99.8% icariin conversion with a 97.8% yield of icariside I, whereas free Rha R783A required 6 h to reach a comparable conversion level (99.8% conversion and 97.5% yield). The enhanced long-term catalytic performance of Rha R783A@MOF may be related to the protective effect of the MOF under the reaction conditions. In particular, 1% (*v*/*v*) DMSO was added to improve the solubility of poorly water-soluble icariin. Although short-term exposure to DMSO enhanced the activity of both free and immobilized Rha R783A (Figure 6b), prolonged exposure to the organic cosolvent during the catalytic reaction could compromise the stability and activity of free R783A. In contrast, encapsulation within the MOF provided a confined protective environment that helped preserve enzyme activity. Notably, the control experiment demonstrated that PVP itself had no significant effect on the catalytic activity or stability of free Rha R783A under identical reaction conditions (Figure S6).

Reusability is an important advantage of enzyme encapsulation systems, as it reduces enzyme consumption and improves the practical applicability of biocatalytic processes. Therefore, the reusability of Rha R783A@MOF was evaluated through consecutive catalytic cycles, and the results were shown in Figure 7b. Rha R783A@MOF exhibited excellent operational stability during repeated reactions, maintaining an icariside I yield above 90% after six consecutive cycles. After eight cycles, the catalyst still retained high activity with an icariside I yield of 88.9%, while the yield gradually decreased to 72.8% after ten cycles. A gradual decrease in catalytic performance was observed in subsequent cycles, primarily due to partial enzyme leaching from the MOF matrix, as evidenced by increase in protein concentration in the supernatant at the 10th cycle (Figure S7), along with cumulative activity loss over prolonged reaction times, which may be associated with the mechanical shear forces exerted by orbital shaking during repeated batch operations. Despite the decline in catalytic activity, the morphology of Rha R783A@MOF remained largely intact after 10 cycles of reuse (Figure S8). Nevertheless, Rha R783A@MOF retained more than 70% of its catalytic performance after ten consecutive cycles at 60 °C, demonstrating excellent operational stability under relatively harsh reaction conditions. Compared with previously reported enzyme-MOF systems, Rha R783A@MOF showed superior recyclability. For example, α-L-rhamnosidase immobilized on Fe_3_O_4_/MIL-101(Cr) retained only about 30% residual activity after 9 cycles [38], and the enzyme immobilized on Fe_3_O_4_ nanoparticles retained 61.34% after only 5 cycles [39], Even the Ce-BTC immobilized α-L-rhamnosidase reported by Peng et al. retained 80% residual activity after five consecutive cycles [27]. In contrast, Rha R783A@MOF maintained an icariside I yield of 93.3% after five cycles, and retained more than 70% of its catalytic performance after ten consecutive cycles at 60 °C, highlighting the potential of this Cu-based MOF encapsulation strategy for sustainable biocatalytic applications.

## 3. Materials and Methods

### 3.1. Materials and Reagents

p-Nitrophenol (pNP), p-nitrophenyl-α-L-rhamnopyranoside (pNPR), icariin, and icariside I were obtained from Macklin (Shanghai, China). Isopropyl β-D-1-thiogalactopyranoside (IPTG) and kanamycin, used for recombinant protein expression, were also purchased from the same supplier. Polyvinylpyrrolidone (PVP, average molar weight 30 K) was purchased from Yuanye (Shanghai, China), and p-aminobenzoic acid (PABA) was obtained from Aladdin (Shanghai, China). All other chemicals were of analytical grade and sourced from commercial suppliers. Ultrapure water was used for preparing all aqueous solutions, and all reagents were employed directly without additional purification.

### 3.2. Expression and Purification of α-L-Rhamnosidase R783A, and Activity Determination

The R783A mutant of α-L-rhamnosidase (Rha R783A) was derived from the wild-type enzyme of *Dictyoglomus thermophilum* (GenBank: WP_012548615). A codon-optimized synthetic gene was obtained from Genscript (Nanjing, China), and the R783A mutation was introduced by site-directed mutagenesis. The mutant gene was inserted into the pET-28a vector and overexpressed in *E. coli* BL21(DE3) with an N-terminal His-tag. Subsequent expression, purification, and activity assays were performed as follows [18]. Cells were grown in LB medium containing 50 μg/mL kanamycin at 37 °C to an OD600 of 0.6–0.8, and expression was induced with 0.1 mM IPTG at 20 °C for 20 h. Harvested cells were disrupted by sonication, and the clarified crude extract was subjected to Ni-NTA affinity chromatography. Protein purity was checked by SDS-PAGE, and concentration was quantified with the Bradford method [40] using BSA as a standard. The activity of Rha R783A was assayed spectrophotometrically using pNPR as substrate. The reaction mixture (1.0 mL) contained 2 mM pNPR, and appropriately diluted enzyme solution in Tris-HCl buffer (100 mM, pH 8.0). The absorbance increase at 405 nm due to pNP release was continuously recorded for 3 min, and the initial linear slope was used to calculate the reaction rate. One unit (U) was defined as the amount of enzyme liberating 1 µmol of pNP per minute. All assays were performed in triplicate.

### 3.3. Synthesis of MOF

The Cu-based metal–organic frameworks (Cu-MOF) were constructed via a co-precipitation method using metal ions and organic ligands as building blocks. Briefly, 50 mM copper acetate was dissolved in deionized water with the aid of ultrasonication, while 12.5 mM PABA was dissolved in acetate buffer (100 mM, pH 6.0). The two solutions were then slowly mixed under continuous stirring at room temperature (25 °C) for 8 h. The resulting green precipitate was collected by centrifugation, washed three times with deionized water, and stored at 4 °C for further use.

### 3.4. Preparation of Rha R783A@MOF Biocomposite

The Rha R783A@MOF biocomposite was fabricated via an in situ encapsulation strategy during MOF synthesis. Briefly, 50 mM copper acetate was dissolved in deionized water with the aid of ultrasonication. Subsequently, the purified Rha R783A enzyme and PVP were dissolved in acetate buffer (100 mM, pH 6.0) and added to the copper acetate solution. Meanwhile, 12.5 mM PABA was separately dissolved in the same acetate buffer (100 mM, pH 6.0). The two solutions were then slowly mixed and stirred continuously at room temperature (25 °C) for 8 h to allow simultaneous formation of the MOF and enzyme encapsulation. The resulting biocomposite was collected by centrifugation, washed three times with deionized water to remove unbound enzyme and residual reagents, and stored at 4 °C for further use.

To prepare the physical adsorption control, an equivalent amount of pure MOF and Rha R783A protein were physically mixed under the same temperature and buffer conditions as those used for the preparation of the Rha R783A@MOF biocomposite, continuously stirred at room temperature (25 °C) for 8 h, and then vacuum freeze-dried. The resulting sample was denoted as Rha R783A&MOF.

To accurately quantify the amount of enzyme physically adsorbed on the external surface, protein quantification was performed after SDS-mediated desorption. Specifically, 2.0 mg of Rha R783A@MOF and Rha R783A&MOF were dispersed in 2 mL 1% (*w*/*v*) SDS solution at room temperature (25 °C) for 5 min. The supernatant was then collected by centrifugation and appropriately diluted to reduce the SDS concentration below the interference threshold of the Bradford assay. Protein concentration was determined using the Bradford assay with bovine serum albumin (BSA) as the standard. The SDS concentration and dilution factor were optimized to avoid interference with the Bradford reagent.

### 3.5. Optimization of Immobilization Conditions

The immobilization conditions for Rha R783A@MOF were optimized by systematically evaluating the effects of enzyme amount, PVP dosage, and immobilization time on enzyme loading and activity recovery. First, the effect of enzyme concentration was investigated by adding different concentrations of Rha R783A (0.1–2 mg/mL) during the construction of the Rha R783A@MOF biocomposite. Subsequently, the PVP dosage was optimized by adding varying amounts of PVP (0.1–2 mg/mL) using the optimal enzyme amount under the same immobilization conditions. Finally, the effect of immobilization time was evaluated within the range of 4–12 h using the optimized amounts of R783A and PVP. After each immobilization experiment, the mixture was centrifuged, and the amount of unbound protein in the supernatant was quantified using the Bradford assay.

Enzyme loading (mg/g) and encapsulation efficiency (%) was calculated as follows:

Enzyme loading (mg/g) = (M_0_ − M_1_)/M (1)

Encapsulation efficiency (%) = (M_0_ − M_1_)/M_0_ (2)

where M_0_ and M_1_ are the initial and unbound enzyme masses, and M is the Rha R783A@MOF biocomposite mass.

Relative activity is defined as the ratio of the catalytic activity of each formulation to that of the formulation with the highest activity, expressed as a percentage. All optimizations were performed in triplicate, and the optimal parameters were used for subsequent experiments.

### 3.6. Characterization of Materials

The morphology and microstructure of the synthesized MOF and Rha R783A@MOF biocomposite were examined using scanning electron microscopy (SEM) (Quattro S, Thermo Fisher Scientific, Waltham, MA, USA) coupled with an energy-dispersive X-ray spectrometer (EDS) (Elect Plus, EDAX, Mahwah, NJ, USA) for elemental composition analysis. The crystal structures of the materials were characterized by X-ray diffraction (XRD) on a Bruker D8 ADVANCE diffractometer (Karlsruhe, Germany) using Cu Kα radiation (λ = 1.5418 Å) at 40 kV and 40 mA. Diffraction patterns were recorded over a 2*θ* range of 5–80° with a scanning 10° min^−1^. FTIR spectra were recorded using a Thermo Scientific Nicolet iS20 FTIR spectrometer equipped with an iN5 infrared microscope. The samples were ground with anhydrous KBr and pressed into pellets, and spectra were recorded in the wavenumber range of 400–4000 cm^−1^. Thermogravimetric analysis (TGA) was performed on a TGA550 thermogravimetric analyzer (TA Instruments, New Castle, DE, USA) to evaluate the thermal stability of the materials. Approximately 5–10 mg of each sample was heated from 25 to 600 °C under a nitrogen atmosphere at a heating rate of 10 °C min^−1^.

### 3.7. Biochemical Characterization of Free and Immobilized R783A

#### 3.7.1. Effects of Temperature and pH on Enzyme Activity

The catalytic properties of free Rha R783A and Rha R783A@MOF were evaluated using pNPR as the substrate under standard assay conditions. The optimal pH was then assessed at 60 °C at a pH range of 3.0–9.0 using different buffer systems: citrate–phosphate buffer (100 mM, pH 3.0–6.0), phosphate buffer (PB, 100 mM, pH 6.0–8.0), and Tris-HCl buffer (100 mM, pH 8.0–9.0). To determine the optimal reaction temperature, the activity of both free and immobilized enzymes was measured at various temperatures ranging from 40 to 90 °C in Tris-HCl buffer (100 mM, pH 8.0). The activity measured at the optimal temperature and pH was defined as 100%, and the relative activities at other conditions were calculated accordingly. All experiments were performed in triplicate, and the data were presented as mean ± standard deviation (SD).

#### 3.7.2. pH Tolerance, Thermostability, and Storage Stability

The pH tolerance of free Rha R783A and Rha R783A@MOF was evaluated by pre-incubating the enzymes in different buffer systems (100 mM) over a pH range of 3.0–9.0 at 25 °C for 2 h. Thermostability was assessed by incubating the free and immobilized enzymes at 60 °C) in Tris-HCl buffer (100 mM, pH 8.0) for 2 h. After incubation, the residual activity of each sample was measured under standard assay conditions and expressed as a percentage of the initial activity measured immediately before pre-incubation, which was defined as 100%.

For storage stability, both free and immobilized Rha R783A were stored in Tris-HCl buffer (100 mM, pH 8.0) at 4 °C for 30 days. At regular intervals (0, 3, 6, 9, 12, 15, 20, 25, and 30 days), aliquots were taken and their residual activities were measured under standard assay conditions. The initial activity (day 0) was defined as 100%. All experiments were conducted in triplicate, and the results were expressed as mean ± standard deviation.

#### 3.7.3. Effects of Metal Ions and Organic Solvents

The tolerance of free Rha R783A and Rha R783A@MOF to various metal ions and organic solvents was systematically evaluated by measuring their residual activity after treatment. For metal ion tolerance, the enzymes were incubated with 1 mM of various metal ions and chemical reagents, including Na^+^, K^+^, Ca^2+^, Cu^2+^, Mg^2+^, Zn^2+^, Mn^2+^, Fe^2+^, Fe^3+^, dithiothreitol (DTT), and ethylenediaminetetraacetic acid (EDTA) in Tris-HCl buffer (100 mM, pH 8.0). The enzyme sample without any additive was used as the control (100%). For organic solvent tolerance, the enzymes were incubated with various organic solvents (10%, *v*/*v*), including methanol, ethanol, dimethyl sulfoxide (DMSO), and acetonitrile (ACN), at final concentrations of 10% (*v*/*v*) in Tris-HCl buffer (100 mM, pH 8.0). The residual activity was then measured under standard assay conditions, and the activity of the sample without any organic solvent was taken as the control (100%). All experiments were performed in triplicate, and results were expressed as mean ± standard deviation.

#### 3.7.4. Kinetic Parameters

The catalytic kinetics of free Rha R783A and Rha R783A@MOF were investigated using pNPR as the substrate. Initial reaction rates were monitored by measuring the absorbance increase at 405 nm for 3 min under the optimized assay conditions, and the initial linear slope was used to calculate *V*_0_. The kinetic constants *K*_m_ and *V*_max_ were determined by fitting the experimental data to the Michaelis–Menten equation:

*V*_0_ = *V*_max_ × [S]/(*K*_m_ + [S]) (3)

*k*_cat_ = *V*_max_/[E]tot (4)

where the *V*_0_ is the initial catalytic reaction rate, *V*_max_ is the maximum catalytic reaction rate, [S] is the initial substrate concentration, and *K*_m_ is the Michaelis–Menten constant. [E]total refers to the total enzyme concentration. All experiments were carried out in triplicate, and data were presented as mean ± standard deviation.

### 3.8. Application of Rha R783A@MOF in Biocatalytic Reactions and Its Reusability

The biocatalytic performance of Rha R783A@MOF was evaluated by its ability to convert icariin to icariside I under the optimized reaction conditions. The enzymatic reactions were carried out in 2 mL of Tris-HCl buffer (100 mM, pH 8.0) containing 5 mM icariin and 0.5 U/mL of Rha R783A@MOF, with 1% (*v*/*v*) DMSO added to improve the solubility of icariin. The reaction mixtures were incubated at 60 °C with orbital shaking at 180 rpm. Aliquots were withdrawn at regular time intervals and the reaction was immediately terminated by heating at 100 °C for 5 min. The samples were then centrifuged to remove the immobilized enzyme, and the supernatant was filtered through a 0.22 μm membrane prior to HPLC analysis. For comparison, free Rha R783A at an equivalent enzyme activity (0.5 U/mL) was tested under the same conditions.

Quantitative analysis of icariin and icariside I was performed on a reversed-phase HPLC system (Shimadzu LC-16AT, Kyoto, Japan) equipped with a ZORBAX Eclipse XDB-C18 column (4.6 × 250 mm, 5 μm; Agilent, Santa Clara, CA, USA), according to previously reported methods with minor modifications [41]. The mobile phase consisted of acetonitrile and 0.1% formic acid in water at 1 mL/min, with UV detection at 270 nm. The conversion was defined as the percentage of substrate consumed relative to the initial concentration, while the yield was expressed as the percentage of the measured product amount relative to the theoretical amount based on the initial substrate concentration.

The reusability of Rha R783A@MOF was assessed under the optimized biocatalytic reaction conditions described above. Briefly, after each reaction cycle, the Rha R783A@MOF biocomposite was recovered by centrifugation at 8000× *g* for 10 min, washed three times with Tris-HCl buffer (100 mM, pH 8.0) to remove residual substrate, and then reintroduced into a fresh reaction mixture (2 mL of Tris-HCl buffer containing 5 mM icariin) for the next cycle at 60 °C with orbital shaking at 180 rpm. The supernatant was carefully separated and subjected to HPLC analysis as described above. The protein concentration in the supernatant was determined using the Bradford assay to monitor enzyme leaching from the immobilized biocatalyst. The recovered Rha R783A@MOF was washed three times with fresh Tris-HCl buffer (100 mM, pH 8.0) to remove residual substrate and products, and then resuspended in fresh reaction medium containing 5 mM substrate for the subsequent cycle.

## 4. Conclusions

In this study, an α-L-rhamnosidase mutant R783A (Rha R783A) was successfully encapsulated within a MOF via a one-pot co-precipitation strategy, constructing an efficient biocatalytic system for the conversion of icariin into icariside I. Comprehensive characterization by SEM, XRD, FTIR spectroscopy, and TGA confirmed the successful encapsulation of Rha R783A within the MOF carrier. The encapsulation strategy improved the operational stability of R783A, including good pH adaptability across pH 6.0–8.0, thermal stability (>90% residual activity after incubation at 60 °C for 2 h), and storage stability (86.4% residual activity after 30 days). Notably, Rha R783A@MOF efficiently catalyzed the biotransformation of icariin, achieving 99.8% conversion and 97.8% yield of icariside I. Furthermore, the encapsulated biocatalyst maintained an icariside I yield of 88.9% after eight consecutive reuse cycles, demonstrating excellent recyclability and operational stability under high-temperature reaction conditions (60 °C). These findings demonstrate that Rha R783A@MOF integrates high catalytic efficiency with enhanced stability and reusability, highlighting its potential as a robust biocatalytic platform for the sustainable conversion of poorly soluble natural products.

## Figures and Tables

**Figure 1:**
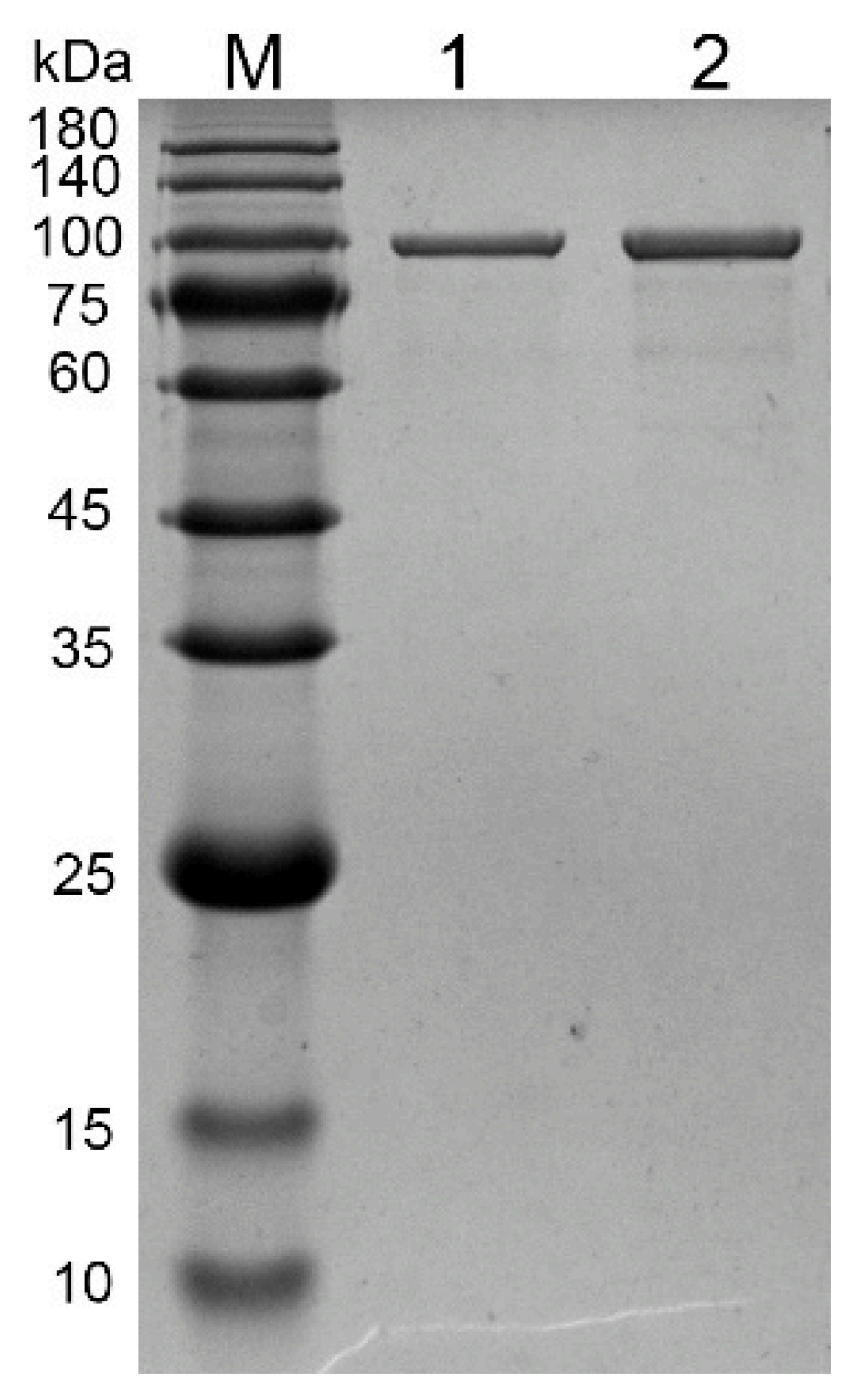
SDS-PAGE analysis of α-L-rhamnosidase and its mutant R783A: lane M, molecular weight standards; lane 1, the wild-type enzyme; lane 2, the purified mutant R783A protein.

**Scheme 1:**
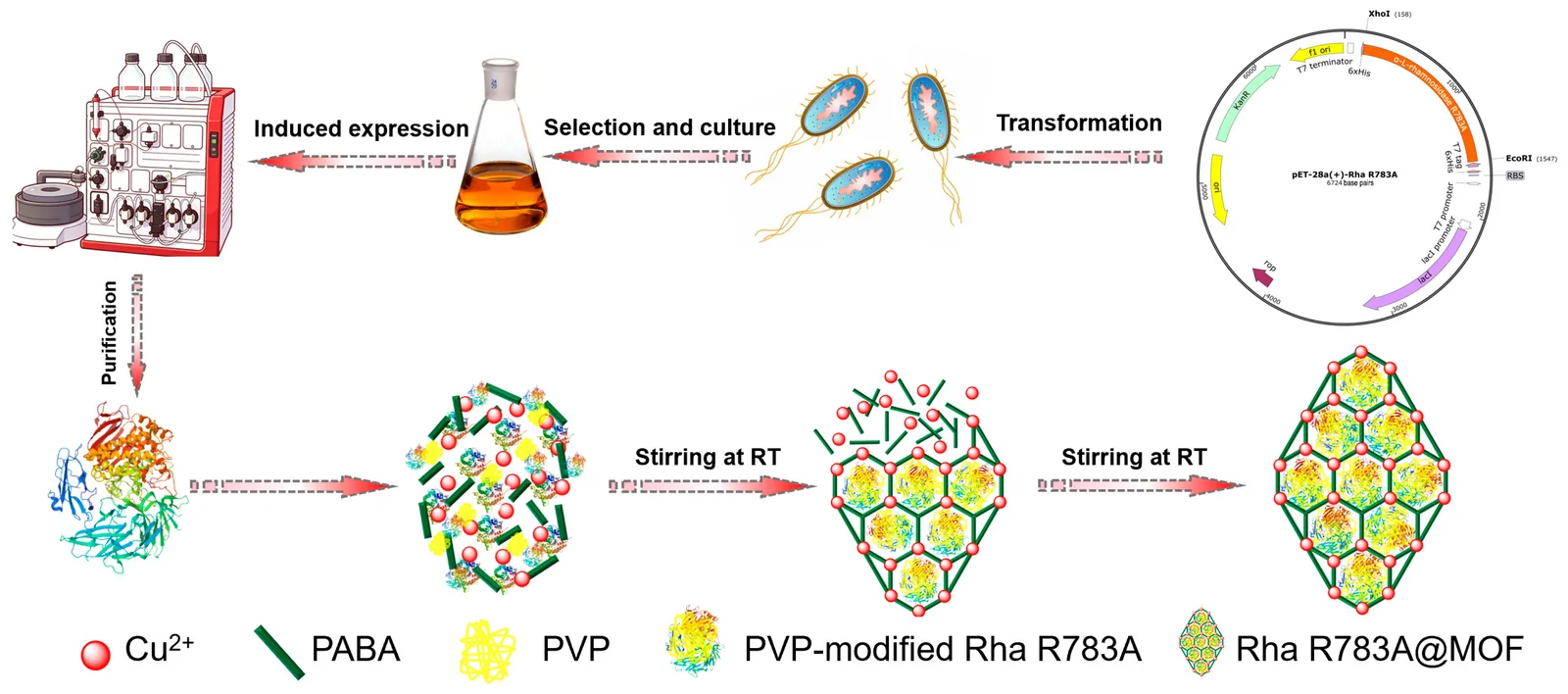
Schematic illustration of the preparation of the Rha R783A@MOF composite via one-pot co-precipitation. RT: room temperature (25 °C).

**Figure 2:**
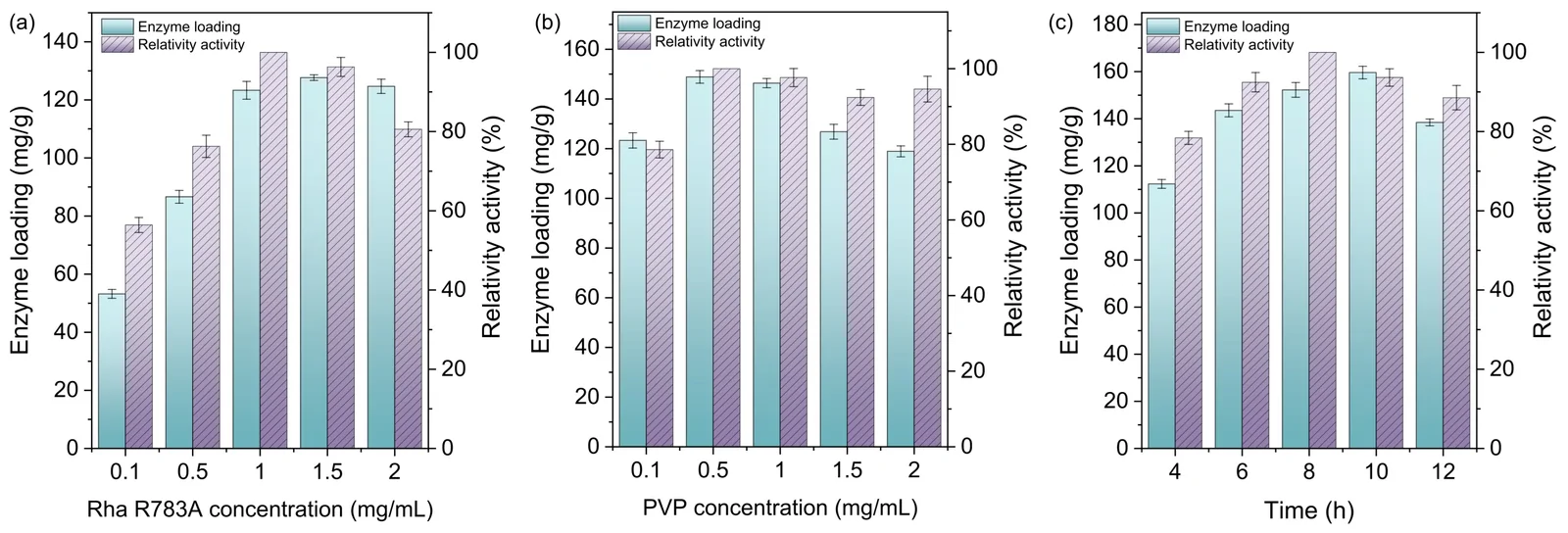
Effects of Rha R783A concentration (**a**), PVP concentration (**b**), and immobilization time (**c**) on the immobilization efficiency of Rha R783A@MOF. The immobilization efficiency was evaluated in terms of enzyme loading (mg/g) and relative activity (%). Error bars represent means ± SD (*n* = 3).

**Figure 3:**
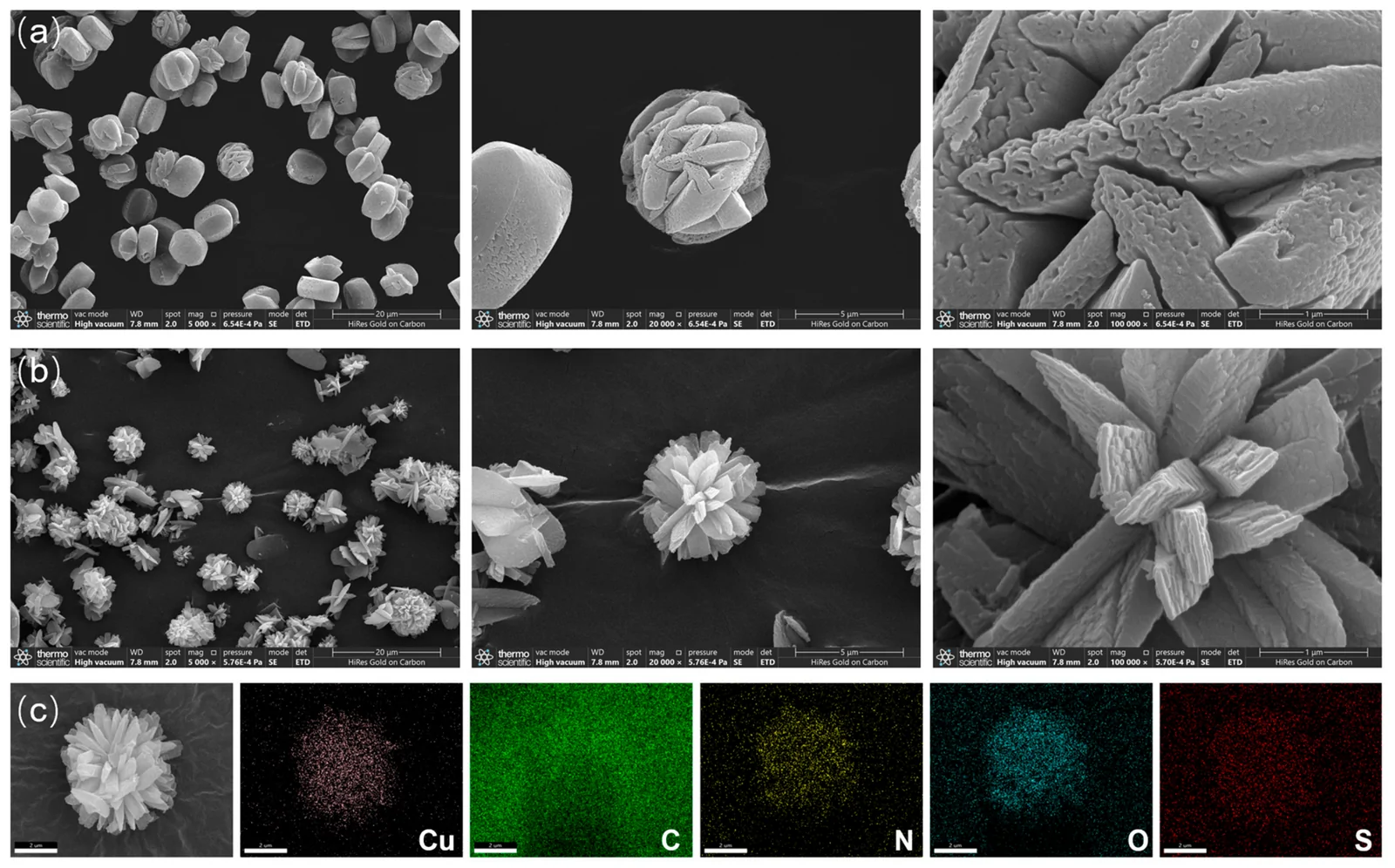
SEM images of the MOF (**a**) and Rha R783A@MOF (**b**), and SEM image with corresponding EDS mapping for Rha R783A@MOF (scale: 2 µm) (**c**).

**Figure 4:**
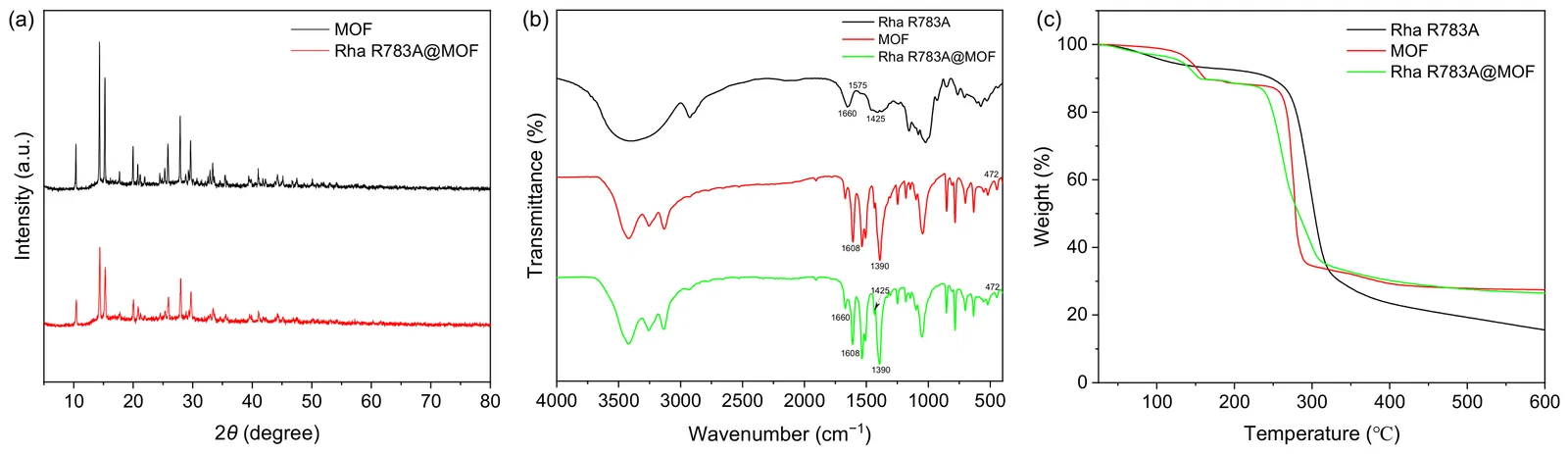
Structural and thermal characterization of MOF before and after Rha R783A immobilization: XRD patterns (**a**), FTIR spectra (**b**), and TGA curves (**c**).

**Figure 5:**
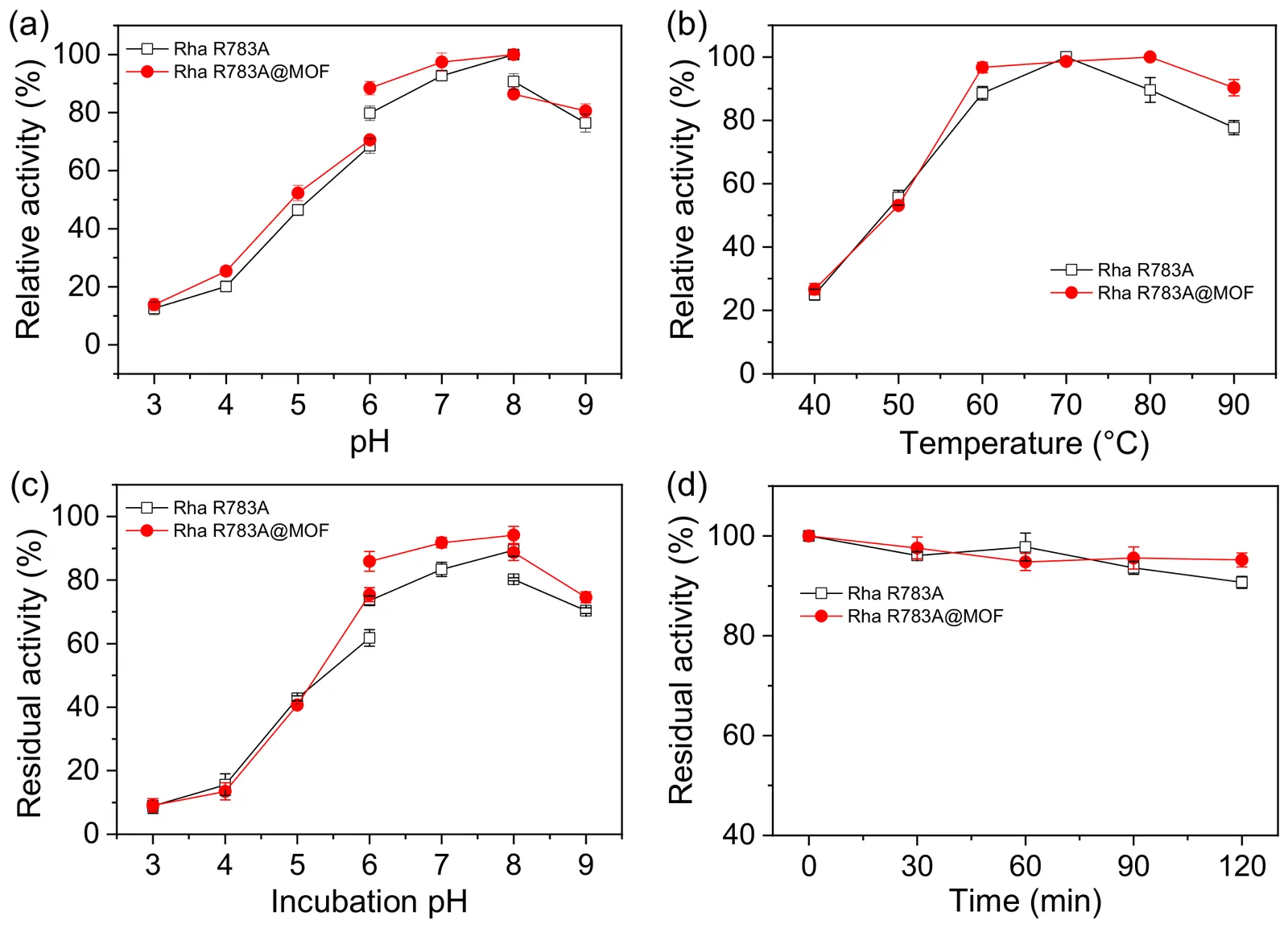
Effects of pH and temperature on the catalytic activity and stability of free Rha R783A and Rha R783A@MOF. (**a**) Optimal reaction pH, (**b**) optimal reaction temperature, (**c**) pH stability, and (**d**) thermostability at 60 °C. All data are presented as the mean ± standard deviation (SD) from three independent replicates (*n* = 3).

**Figure 6:**
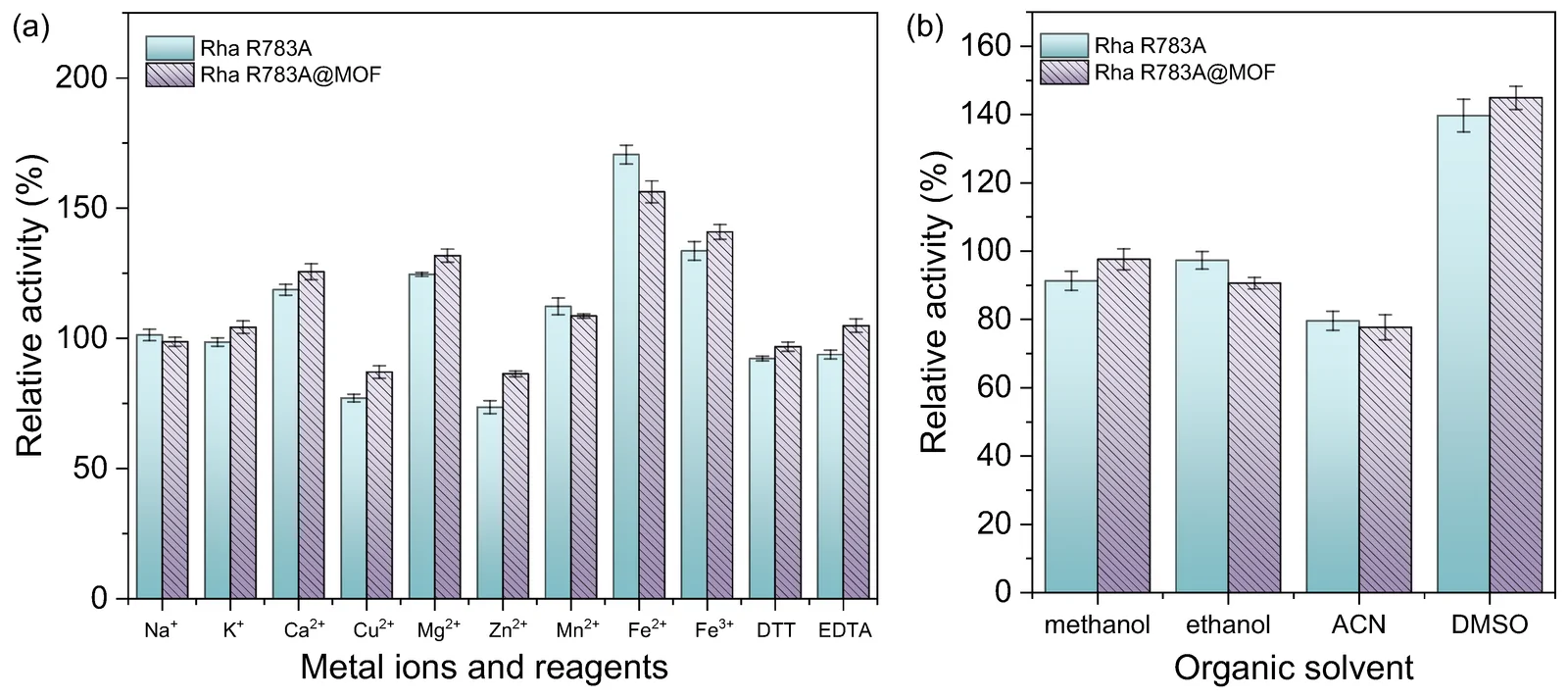
Effects of various metal ions and reagents (**a**) and organic solvents (**b**) on the relative activity of free R783A and Rha R783A@MOF. The activity of the untreated sample (without additives) was defined as 100%. The concentrations of metal ions and reagents were 1 mM, and organic solvents were added at final concentrations of 10% (*v*/*v*). Error bars represent the mean ± SD from three independent replicates (*n* = 3).

**Figure 7:**
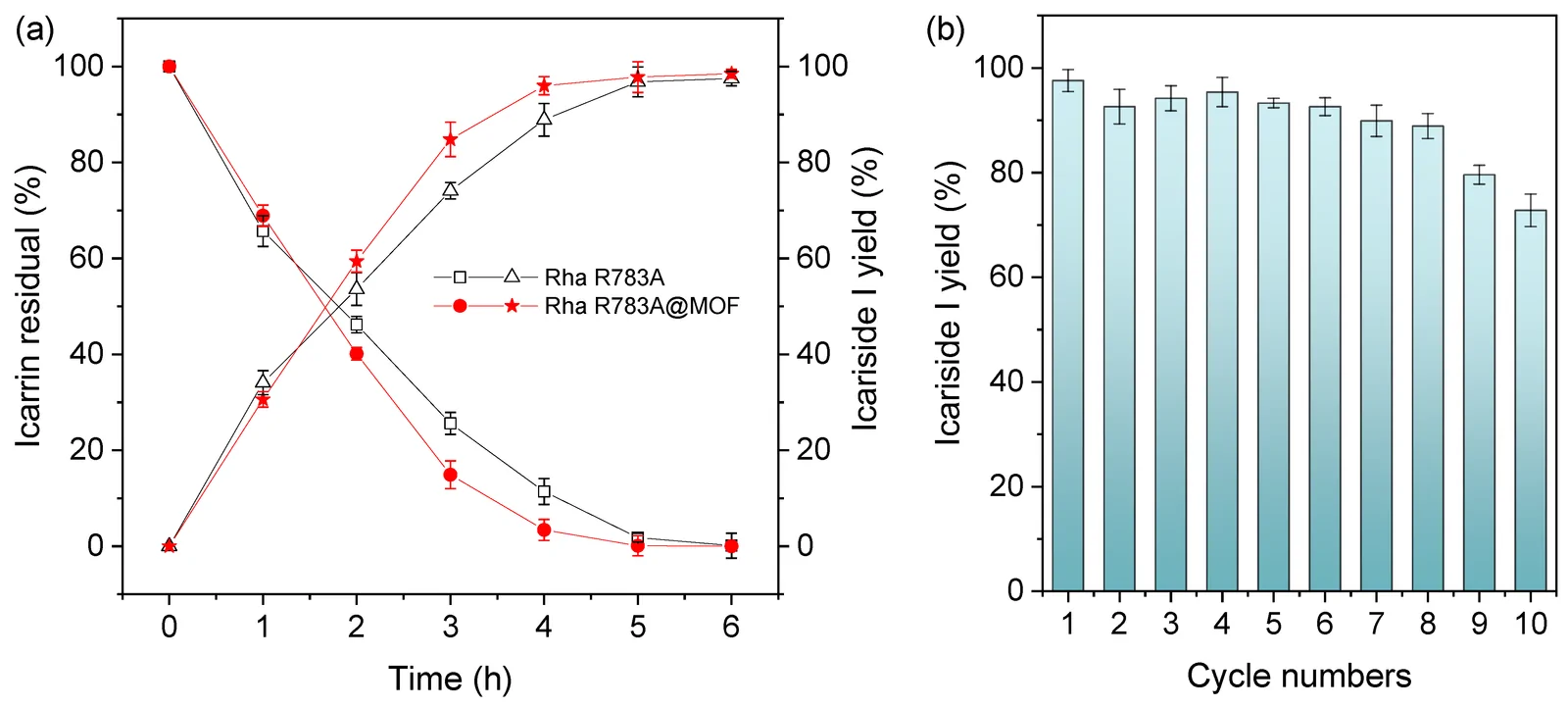
(**a**) Time-dependent conversion of icariin into icariside I catalyzed by free Rha R783A and Rha R783A@MOF. (**b**) Recyclability of Rha R783A@MOF for ten consecutive cycles. Error bars represent means ± SD (*n* = 2).

**Table 1:** Apparent kinetic parameters of free Rha R783A and Rha R783A@MOF.

Enzyme	*V*_max_ (mM s^−1^)	*K*_m_ (mM)	*k*_cat_, ^app^(s^−1^)	*k*_cat_/*K*_m_ (s^−1^ mM^−1^)
Rha R783A	2.71 ± 0.028	0.41 ± 0.022	17.31 ± 0.21	42.22 ± 2.32
Rha R783A@MOF	2.82 ± 0.016	0.48 ± 0.018	23.05 ± 0.18	48.02 ± 1.84

The experimental data are presented as mean ± SD (*n* = 3).

## Data Availability

The original contributions presented in this study are included in the article/Supplementary Material. Further inquiries can be directed to the corresponding author.

## Supplementary Materials

The following supporting information can be downloaded at: https://www.mdpi.com/article/10.3390/molecules31203615/s1, Table S1. Secondary structures of free Rha R783A and Rha R783A@MOF within the amide I region. Figure S1. (a) Encapsulation efficiency of Rha R783A@MOF and Rha R783A&MOF, and (b) residual protein of Rha R783A@MOF and Rha R783A&MOF after SDS washing. Figure S2. Storage stability of the free Rha R783A and Rha R783A@MOF at 4 °C. Figure S3. The relative activity of Rha R783A@MOFcompared with free Rha R783A. Table S2. Effects of Cu^2+^ and EDTA on the activity of Rha R783A enzyme. Figure S4. Michaelis–Menten plots of the free Rha R783A (a) and Rha R783A@MOF (b). Figure S5. HPLC spectrogram for analysis of icariside I from icariin. Figure S6. Catalytic control experiment: Time-dependent conversion of icariin to icariside I catalyzed by free Rha R783A and free Rha R783A in the presence of 0.5 mg/mL PVP. Figure S7. Concentration of leached protein in the reaction supernatant during 10 cycles of icariin bioconversion catalyzed by Rha R783A@MOF. Figure S8. SEM images of the Rha R783A@MOF after the 10th catalytic cycle. Figure S9. Uncropped SDS-PAGE gel image of purified α-L-rhamnosidase and its mutant Rha R783A. Table S3. Rha R783A mutant protein sequence.
