# Supplementary material for: One-Pot Encapsulation of a Robust α-L-Rhamnosidase Mutant in Cu-MOF: A Recyclable Designed Biocatalyst for High-Yield Conversion of Icariin to Icariside I
Source: Molecules. 2026 Oct 10;31(20):3615. doi: 10.3390/molecules31203615 (PMC13707456; doi:10.3390/molecules31203615)
Supplement: Supplementary file 1 [file molecules-31-03615-s001.zip › molecules-4564996-supplementary.pdf]

Supplementary Files for Article

One-Pot Encapsulation of a Robust α-L-Rhamnosidase Mutant in Cu-MOF: A Recyclable Designed Biocatalyst for High-Yield Conversion of Icariin to Icariside I

Haoyu Jia *, Tong Yan, Luran Wang and Lingling Song

School of Pharmacy, Henan University of Chinese Medicine, Zhengzhou 450046, China; 15137518359@163.com (T.Y.); 16639293199@163.com (L.W.); 18838221762@163.com (L.S.)

* Correspondence: jiahaoyu@hactcm.edu.cn

Table S1. Secondary structures of free Rha R783A and Rha R783A@MOF within the amide I region.

Figure S1. (a) Encapsulation efficiency of Rha R783A@MOF and Rha R783A&MOF, and (b) residual protein of Rha R783A@MOF and Rha R783A&MOF after SDS washing.

Figure S2. Storage stability of the free Rha R783A and Rha R783A@MOF at 4 °C.

Figure S3. The relative activity of Rha R783A@MOFcompared with free Rha R783A

Table S2. Effects of Cu^{2+} and EDTA on the activity of Rha R783A enzyme.

Figure S4. Michaelis-Menten plots of the free Rha R783A (a) and Rha R783A@MOF (b).

Figure S5. HPLC spectrogram for analysis of icariside I from icariin.

Figure S6. Catalytic control experiment: Time-dependent conversion of icariin to icariside I catalyzed by free Rha R783A and free Rha R783A in the presence of 0.5 mg/mL PVP.

Figure S7. Concentration of leached protein in the reaction supernatant during 10 cycles of icariin bioconversion catalyzed by Rha R783A@MOF.

Figure S8. SEM images of the Rha R783A@MOF after the 10th catalytic cycle.

Figure S9. Uncropped SDS-PAGE gel image of purified α-L-rhamnosidase and its mutant Rha R783A.

Table S3. Rha R783A mutant protein sequence.

Table S1. Secondary structures of free Rha R783A and Rha R783A@MOF within the amide I region.

Enzymes	α-Helix (%)	β-Sheet (%)	β-Turn (%)	Random coil (%)
Rha R783A	32.3	21.6	19.7	26.4
Rha R783A@MOF	30.2	24.4	18.2	27.2

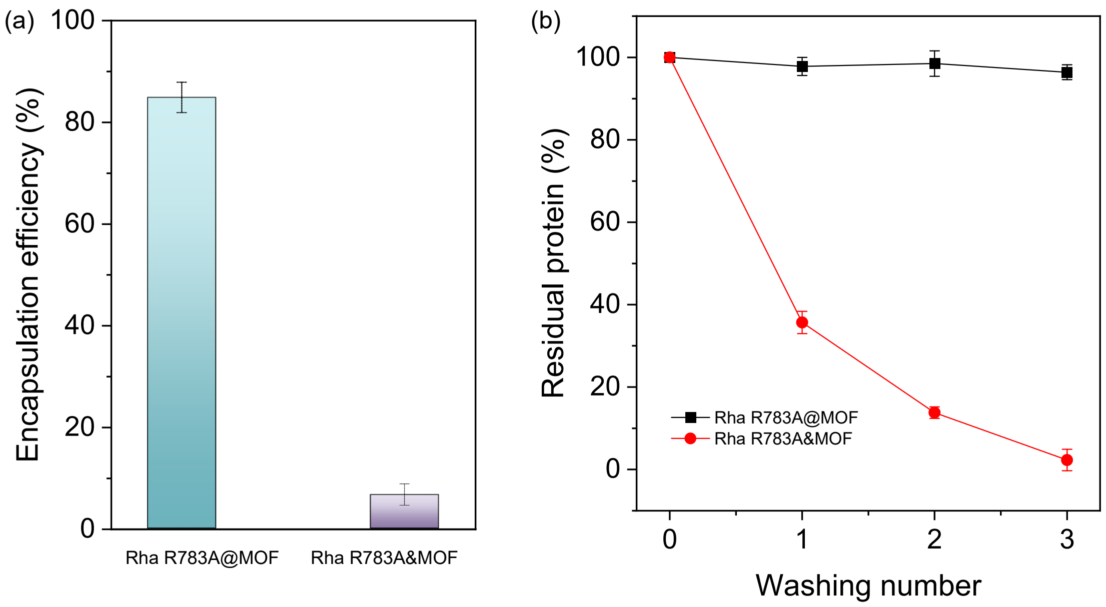

Figure S1. (a) Encapsulation efficiency of Rha R783A@MOF and Rha R783A&MOF, and (b) residual protein of Rha R783A@MOF and Rha R783A&MOF after SDS washing

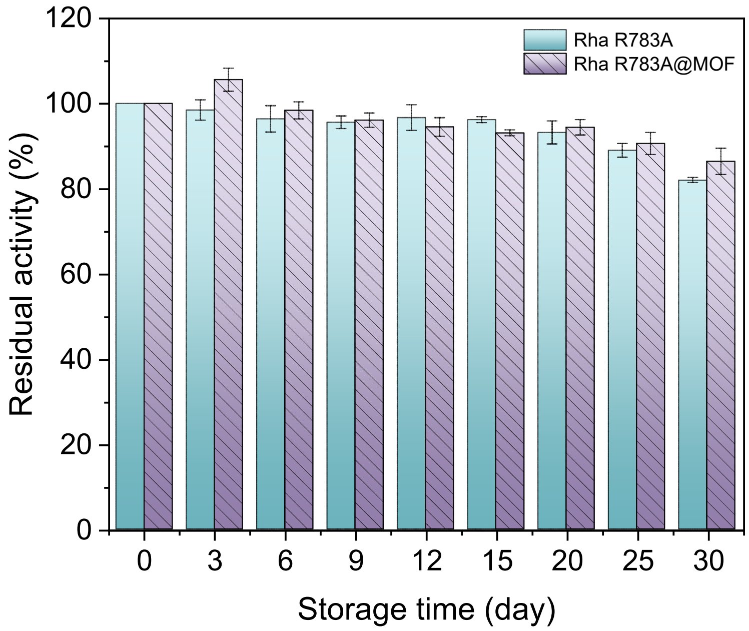

Figure S2. Storage stability of the free Rha R783A and Rha R783A@MOF at 4 °C.

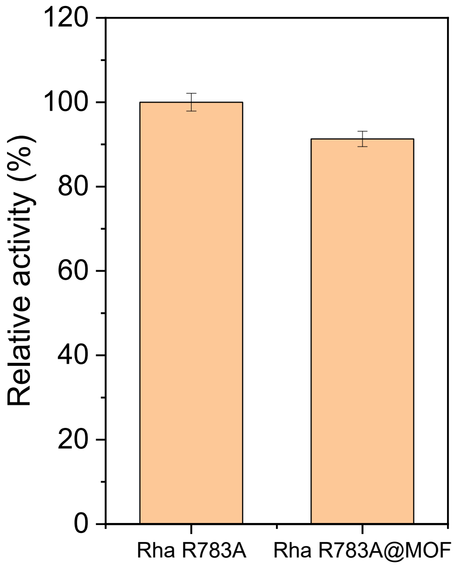

Figure S3. The relative activity of Rha R783A@MOFcompared with free Rha R783A

Table S2. Effects of Cu^{2+} and EDTA on the activity of Rha R783A enzyme.

Entry	Samples	Residual activity (%)
1	Free Rha R783A	100
2	Free Rha R783A+ Cu^{2+}	79 ± 2.1
3*	Free Rha R783A+ Cu^{2+}+ EDTA	89.1 ±1.7
4	Free Rha R783A+ EDTA	94.8 ±1.2

Reaction conditions: The 2.0 mL reaction mixture contained 2 mM pNPR, 1 mM Cu^{2+}, or/and 1.5 mM EDTA, appropriately diluted enzyme in 100 mM Tris-HCl buffer (pH 8.0). The absorbance at 405 nm due to pNP release was continuously recorded for 3 min. *: Entry 3 was measured after adding EDTA to the reaction in entry 2 and incubating for 3 min.

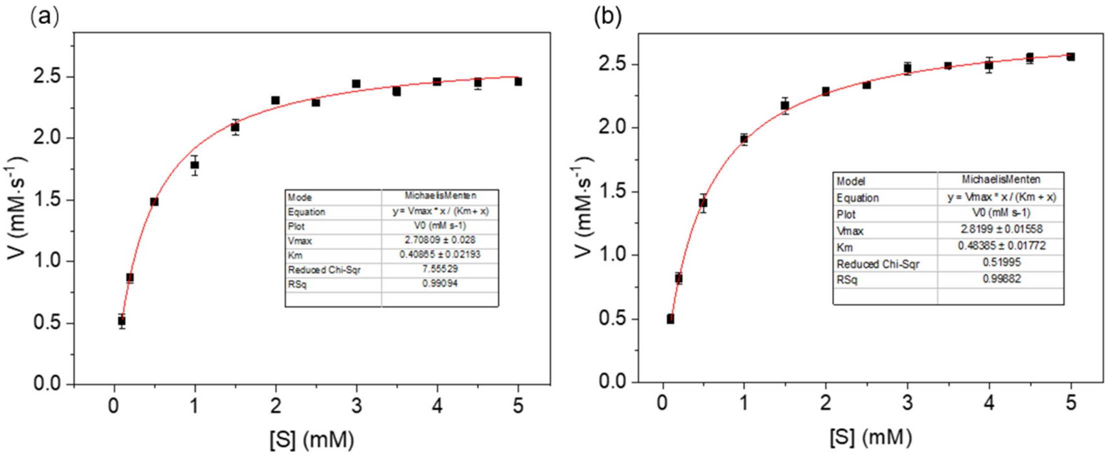

Figure S4. Michaelis-Menten plots of the free Rha R783A (a) and Rha R783A@MOF (b).

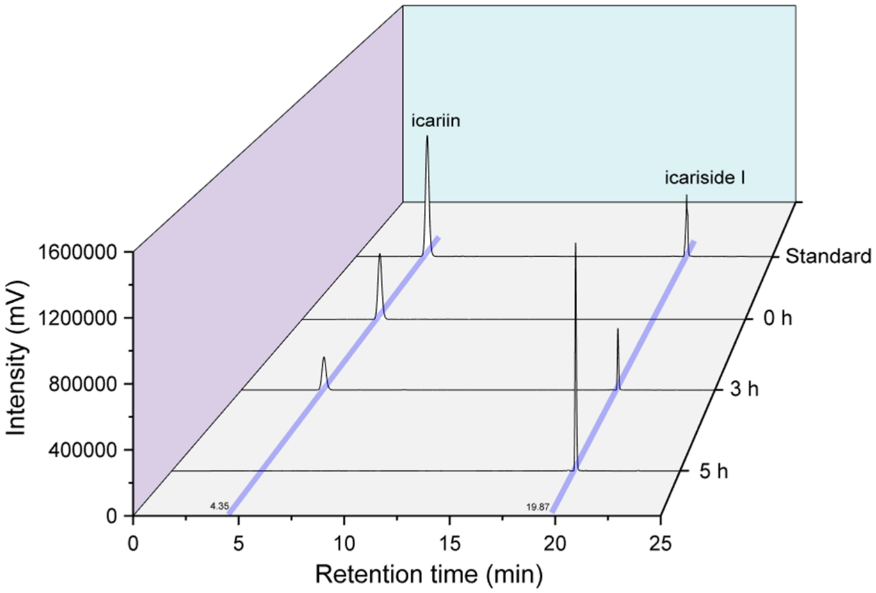

Figure S5. HPLC spectrogram for analysis of icariside I from icariin.

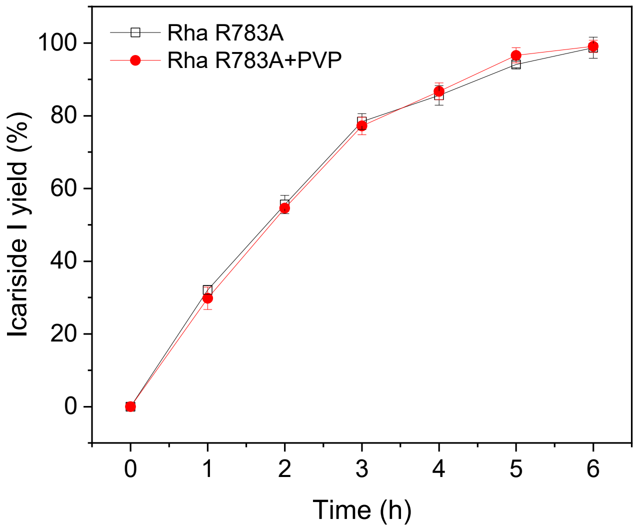

Figure S6. Catalytic control experiment: Time-dependent conversion of icariin to icariside I catalyzed by free Rha R783A and free Rha R783A in the presence of 0.5 mg/mL PVP. performed under the same reaction conditions as those used for free Rha R783A and Rha R783A@MOF.

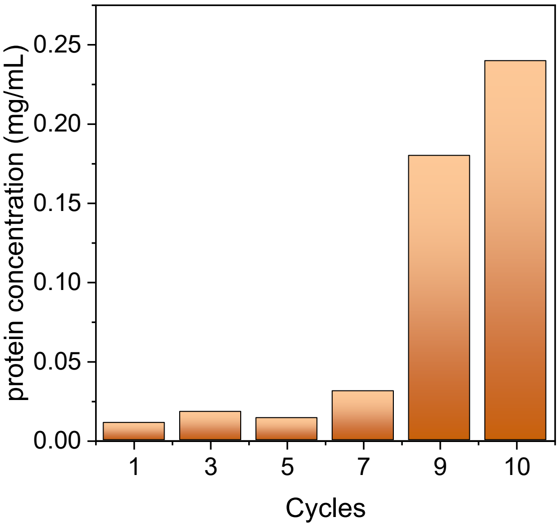

Figure S7. Concentration of leached protein in the reaction supernatant during 10 cycles of icariin bioconversion catalyzed by Rha R783A@MOF.

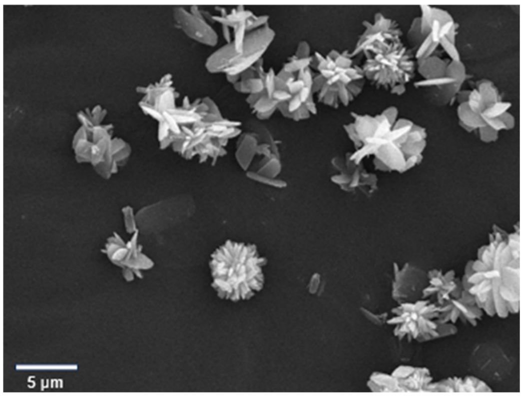

Figure S8. SEM images of the Rha R783A@MOF after the 10th catalytic cycle.

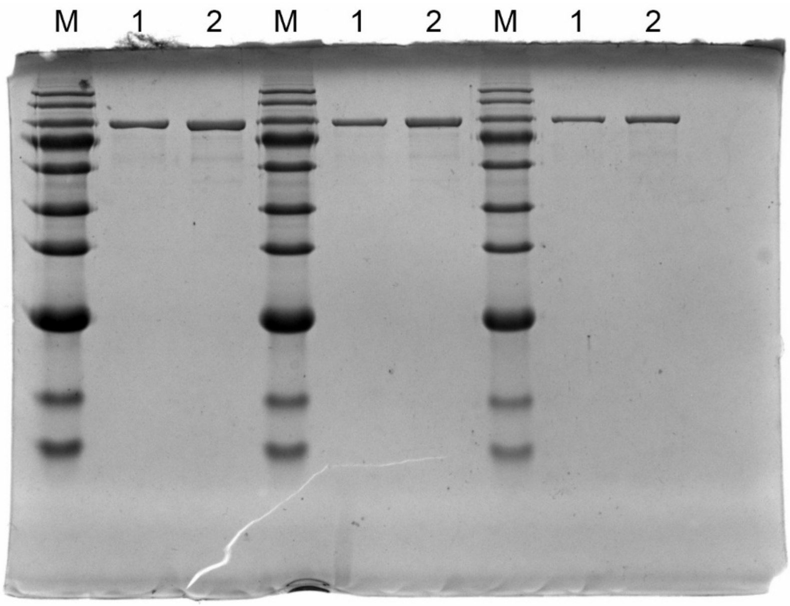

Figure S9. Uncropped SDS-PAGE gel image of purified α-L-rhamnosidase and its mutant Rha R783A.

Table S3. Rha R783A mutant protein sequence.

MKSSNIYSPFDLKCEFTTNPLGVDKKNPIFSWKLRHLEKNEKQTAYQVIVSSSLETINDN<br>IGDVWDTGKVLSSEQVIKYEGKELEPCKVYFWKVRWWDSKDQESPFSVVNTFETGLM<br>NEENWKAKWITKKEHKYEVYSPDGAPFGLNYTIAYAPMFRKSFSISKKIKRARVYIAGL<br>GLYELYINGERIGDRVLDPGQTDYKKRVLYTVYDVSKNIRDGKNAIGVILGNGRYVKEY<br>GYDFPKLIIQVLVEYEDDSIEWIVSDESWKTTYGPITLNSLYHGEIYDGRKEIKGWNLPD<br>FDDSTWENAILAEPPGGKLYSEIYPPIRITKTIKPIKMWSPEPGTYVYDFGQNYTGWIKIK<br>VRTNESGKEIRIRHAELTYEDGTLNYSTNRTALATDVYITKGEGYEEYEPRFTYHGFRYV<br>EILGYPGVPTLEDIEGKVVHTAVESNGEFICSNELINKIHHNIIWGQLSNLMSIPTDCPQ<br>RDERMGWMGDAQLSAEEAIFNFDMIGFYRKYLNDIRDAQKENGSLSDVIPPYWSIYPG<br>DPAWSTAYITIAWYLYQYYGDKYVLEEHYEGFKKYVEFLKKLAPDYIVSFYKYGDWCQ<br>PGTVRPKDNSGELTSTFYFYHDVITLSKIAKLLGKEADYKYYSELADKIKSAFNKKFLKE<br>KAYASIPTELSEENVKALLEKYPEDIKDFLRQQFTILSSLGMFTSQTLNTLPLYLNLVPED<br>KVQDVLKTLLEDIIIRHDYHLDTGIVATRYIFDVLTSYGYDEVAYKIVNQKTYPSFGYMIE<br>EGATTLWEAWEKLTSTGMNSHNHIMFGSVDAWFYRVIAGVRVGEPGWNKIIFEPHPV<br>GDLKYAKARLNTIKGEVEINWQKTENIFSMRISVPVNSEGEVHVPKLFERFVVKEGDNI<br>IYEKKGDLEENEKYIVIRVGSGSYNFYMEKI
